# Characterization of resistance profile (intensity and mechanisms) of *Anopheles gambia*e in three communes of northern Benin, West Africa

**DOI:** 10.1186/s12936-021-03856-2

**Published:** 2021-07-27

**Authors:** Casimir Dossou Kpanou, Hermann W. Sagbohan, Fortuné Dagnon, Germain G. Padonou, Razaki Ossè, Albert Sourou Salako, Aboubakar Sidick, Wilfried Sewadé, André Sominahouin, Patrick Condo, Saadani Hassani Ahmed, Daniel Impoinvil, Martin Akogbéto

**Affiliations:** 1 Centre de Recherche entomologique de Cotonou (CREC), Cotonou, Bénin; 2 Faculté des Sciences et Techniques de l’Université d’Abomey-Calavi, Abomey-Calavi, Bénin; 3 US President’s Malaria Initiative, US Agency for International Development, Cotonou, Bénin; 4 Université Nationale d’Agriculture de Porto-Novo, Porto-Novo, Bénin; 5 US President’s Malaria Initiative, Centers for Disease Control and Prevention for Disease Control (CDC), Georgia, USA; 6 Present Address: Bill & Melinda Gates Foundation, Lagos, Nigeria

**Keywords:** Insecticide resistance, Intensity, *Anopheles gambiae*, Pyrethroids, Bendiocarb

## Abstract

**Background:**

The selection and the spread of insecticide resistance in malaria vectors to the main classes of insecticides used in vector control tools are a major and ongoing challenge to malaria vector control programmes. This study aimed to determine the intensity of vector resistance to insecticides in three regions of Benin with different agro-ecological characteristics.

**Methods:**

Larvae of *Anopheles gambiae *sensu lato (*s.l*.) were collected from September to November 2017 in different larval sites in three northern Benin communes: Parakou, Kandi and Malanville. Two to five-day-old, non-blood-fed, female mosquitoes were exposed to papers impregnated with deltamethrin, permethrin and bendiocarb at dosages of 1 × the diagnostic dose, 5 × and 10 × to determine the intensity of resistance in these vectors. Molecular frequencies of the *kdr* L1014F and *ace-1R* G119S insecticide resistance mutations and levels of detoxification enzymes were determined for mosquitoes sampled at each study site.

**Results:**

Resistance to pyrethroids (permethrin and deltamethrin) was recorded in all three communes with mortality rates below 60% using the diagnostic dose (1x). The results obtained after exposure of *An. gambiae* to permethrin 10 × were 99% in Kandi, 98% in Malanville and 99% in Parakou. With deltamethrin 10x, mortality rates were 100% in Kandi, 96% in Malanville and 73% in Parakou. For the diagnostic dose of bendiocarb, suspected resistance was recorded in the communes of Malanville (97%) and Kandi (94%) while sensitivity was observed in Parakou (98%).Using the 10 × dose, mortality was 98% in Kandi, 100% in Malanville and 99% in Parakou. The frequencies of the *kdr* L1014F allele varied between 59 and 83% depending on the sites and species of the *An. gambiae* complex, while the frequency of the *ace-1R* G119S gene varied between 0 and 5%. Biochemical tests showed high levels of oxidase and esterase activity compared to the susceptible colony strain of *An. gambiae *sensu stricto (Kisumu strain).

**Conclusion:**

*Anopheles gambiae* showed a generalized loss of susceptibility to permethrin and deltamethrin but also showed moderate to high intensity of resistance in different regions of Benin. This high intensity of resistance is a potential threat to the effectiveness of vector control.

## Background

Malaria persists as a major public health problem in sub-Saharan Africa [1]. Benin, like most countries in sub-Saharan Africa, continues to bear a heavy burden of malaria. While the prevalence of malaria is 15% in the general population, this rate is even higher among children under five years of age (37.2%) [2]. Vector control is of paramount importance in the fight against this disease. Current vector control tools mainly include long-lasting insecticidal nets (LLINs) and indoor residual spraying (IRS).

However, over the past decade, studies in Benin have reported an expansion of insecticide resistance in malaria vectors [3–6], particularly in *Anopheles gambiae *sensu lato (*s.l*.), to the different insecticide classes. Resistance has been detected in all pyrethroids used in the impregnation of insecticide-treated nets (ITNs). Carbamates, such as bendiocarb and propoxur, used for IRS, have also been shown to have reduced efficacy against *An. gambiae* in the Atacora department [3]. For organophosphates, a decrease in sensitivity of *An. gambiae* to compounds such as fenitrothion has also been observed [3]; however, although no resistance to pirimiphos-methyl has been recorded [7] despite its use in Atacora for IRS from 2013 to 2016, resistance is a dynamic phenomenon and there is a continuous threat of resistance emergence. This has already been shown by authors from other countries [8, 9].

Phenotypic insecticide resistance is assessed using the World Health Organization (WHO) insecticide susceptibility tests [10] and the Centers for Disease Control and Prevention (CDC) bottle bioassay for determining insecticide resistance in vectors [11]. Many vector control programmes screen for resistance using only the diagnostic dose (1 ×) of the insecticides. While these tests provide useful information on the presence or absence of resistance in mosquito populations, they do not provide details on the intensity of insecticide resistance in non-susceptible populations. For example, if two different mosquito populations had mortality rates of 50% at 1 × , but when exposed to 5 × and 10 × , one population achieved 100% mortality, but the other remained at ~ 50%, the 1 × assay would have erroneously led a decision-maker to believe that the populations were ‘equivalent’ in their resistance. In actuality, the behaviour and the receptivity to control of these two mosquito populations would be different when in contact with insecticide-based vector control tools (LLINs or IRS).

This work aimed to measure the intensity of insecticide resistance in malaria vectors from rice- and cotton-growing environments under high agricultural insecticide pressure against crop pests by exposing them to high doses of bendiocarb, deltamethrin and permethrin. This was done to quantify the level of resistance occurring in northern Benin.

Molecular and biochemical tests were also performed to provide a characterization of the mechanisms conferring insecticide resistance in mosquito populations.

## Methods

### Study area

**Fig. 1:**
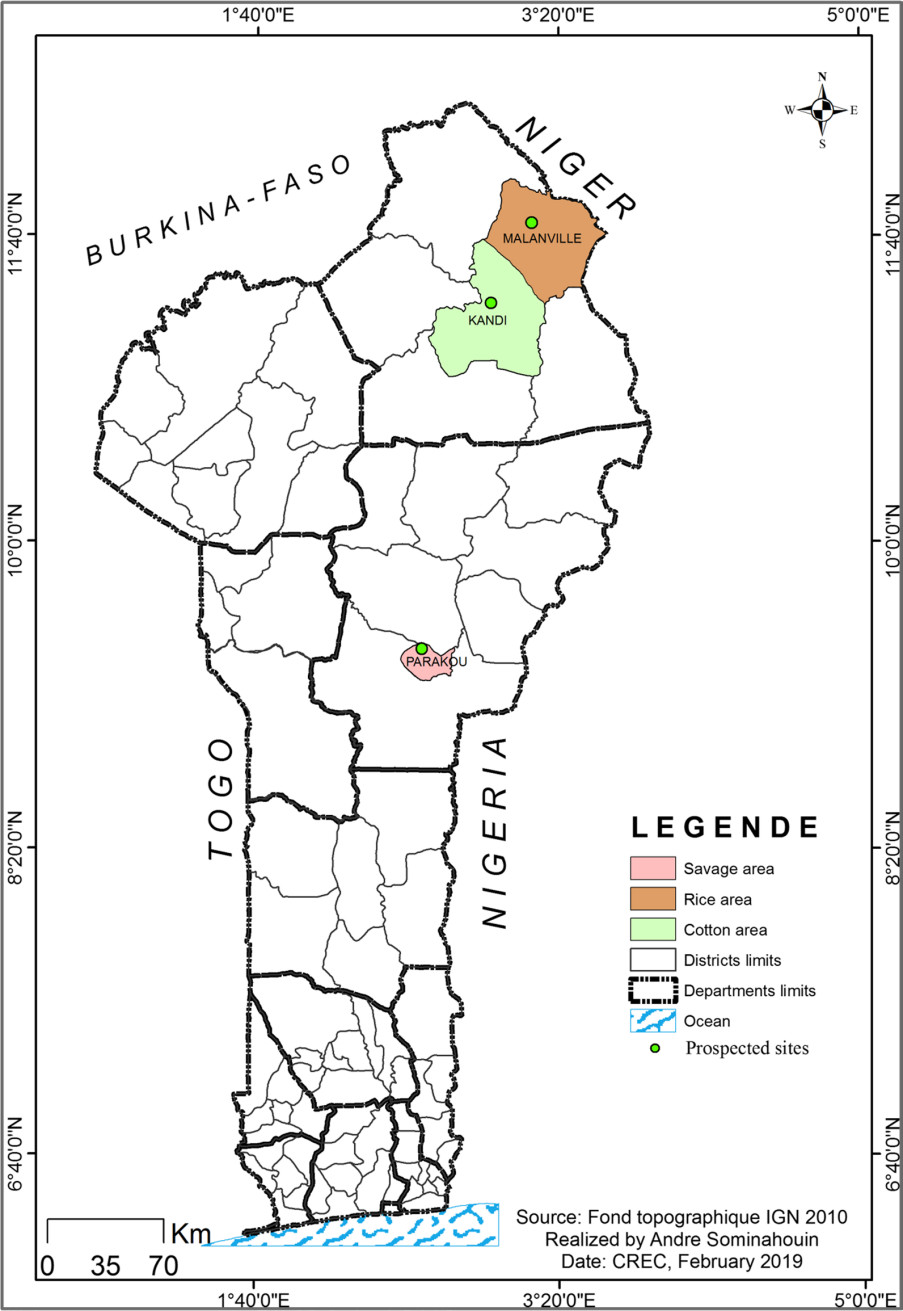
Study area showing the rice-growing commune (Malanville), the cotton-growing commune in Kandi, and the savage area in Parakou

The study was carried out in three communes in northern Benin (Parakou, Kandi, Malanville) between September and November 2017 (Fig. 1). These three communes were selected because of their different agro-ecological characteristics.

The commune of Parakou in the department of Borgou is at an average altitude of 350 m above sea level with a modest relief (i.e., the geographical difference between the highest and lowest elevation point in an area). It covers an area of 441 sq km with a population of 254,254 inhabitants [12]. In Parakou, the climate is humid and tropical (South-Sudanese climate). It is characterized by an annual alternation of a rainy season (May to October) and a dry season (November to April). The lowest temperatures are recorded in December-January. The average annual rainfall is 1,200 mm, with a maximum occurring between July and September. The landcover of Parakou is predominately urban. This town has not conducted an IRS campaign so far, but cotton is widely grown with extensive use of insecticides to control agricultural pests. Also, people frequently use LLINs, aerosol sprays and smoke coils to protect themselves against mosquito bites. There is market gardening (small-scale commercial production of cash crops) occurring along the commune perimeter, which serves as prolific larval habitats for *An. gambiae* on both sides of the city.

The commune of Kandi is located in the centre of the Alibori department in the agro-ecological zone of the cotton basin. It is an area with high cotton production and therefore intensive use of insecticides. These insecticides belong to the class of pyrethroids, carbamates and organophosphates [13]. In addition, this commune benefitted from intradomiciliary spraying based on pirimiphos-methyl in 2017. The Kandi commune covers an area of 3,421 sq km, (i.e., about 13% of the entire department) with a population of 177,683 inhabitants [12]. Kandi has an altitude of 200 to 300 m and is cut by steep valleys, including the Sota valley and the Alibori valley in the east and the west, respectively. The relief is made up of sandstone plateaux cut by the valleys of the Sota and Alibori rivers, which are the two main rivers of the commune. The climate is North-Sudanese with a dry season from November to April and a rainy season from May to October. The average rainfall is between 800 and 1,300 mm per year. The dominant winds are the harmattan in the dry season and the monsoon from April to October. The average relative humidity reaches 80% during the rains but drops to 35% during the dry season. The major sources of water are the tributaries of the Alibori and the Sota [14].

The commune of Malanville, also in the Alibori department, is located in the extreme north of the Republic of Benin. It covers an area of 3,016 sq km, of which 8,000 ha is arable land. Its population is 168,006 inhabitants [12]. Like the rest of the country, the commune of Malanville regularly benefits from LLIN campaigns. Its average altitude is 200 m above sea level. Its climate is of the North-Sudanese type marked by a dry season from November to April. The annual average rainfall recorded is 750 mm. Malanville is crossed longitudinally (east to west) by the Niger River with its tributaries, including the Alibori, the Mékrou and the Sota river, which flood during August and September. The commune of Malanville is an area of high rice production; rice plots serve as larval habitats for *An. gambiae s.l.* The application of tonnes of fertilizers and herbicides applied to the rice plots every year has contributed to the emergence of insecticide resistance in this commune [15].

### Mosquito collection

*Anopheles gambiae* larvae were collected during the study period from different larval sites in the communes of Parakou, Kandi and Malanville using standard dippers and containers. The collected larvae were sorted, kept in labelled jars, and transported to the insectary of the Centre for Entomological Research of Cotonou (CREC) for rearing. Emerging adults from the field larval collections, which were placed in cages, were fed on 10% honey solution and kept at 27 ± 2 °C and relative humidity of 72 ± 5%. Morphologically identified 2 to 5-day-old adult females were used for susceptibility testing to various insecticides and biochemical analyses.

### Susceptibility testing of collected *Anopheles gambiae* to insecticides

Susceptibility tests using WHO tubes were performed according to the WHO protocol [10] with non-blood-fed female *An. gambiae* aged 2–5 days. These mosquitoes were exposed to different doses of WHO-impregnated papers: deltamethrin, permethrin and bendiocarb. Deltamethrin and permethrin are the most common insecticides found on mosquito nets in Benin, and bendiocarb was the insecticide formerly used in some regions of Benin (Oueme and Atacora) from 2008 to 2012 but to which vectors have developed resistance [3]. The doses of the different tested insecticides were: deltamethrin 0.05% (1 ×); deltamethrin 0.25% (5 ×); deltamethrin 0.5% (10 ×); permethrin 0.75% (1 ×); permethrin 3.75% (5 ×); permethrin 7.5% (10 ×); bendiocarb 0.1% (1 ×), 0.5% (5 ×) and 1% (10 ×). The use of higher concentrations (5 × and 10 ×) allows for the determination of the intensity of insecticide resistance in these populations and their ability to tolerate insecticide doses that are higher than the diagnostic dose.

For each dose of insecticide, approximately 20 non-blood-fed female mosquitoes were introduced into each tube lined with insecticide-impregnated paper. For each insecticide and dose, four test tubes lined each with the respective insecticide-impregnated paper were used. For each assay, two control tubes with impregnated paper with insecticide diluent only (i.e., no insecticide) were used; each tube had ~ 20 non-blood-fed female mosquitoes. After 60 min of exposure, the mosquitoes were transferred to observation tubes containing untreated paper, with free access to 10% honey solution. At the end of the tests, living and dead specimens were used for molecular identification of species and determination of resistance mechanisms.

### Identification of *Anopheles gambiae* complex species and molecular characterization of *kdr* L1014F and *ace-1* G119S resistance alleles

Using previously established protocols [16], live and dead mosquitoes from the susceptibility tests from all doses were analysed by PCR to determine the species of the *An. gambiae* complex.

The genotypes of the *kdr* (knock-down resistance) L1014F mutation in the sodium channel associated with resistance to pyrethroid insecticides and the *ace-1* G119S (insensitive acetylcholinesterase**)** mutation associated with resistance to carbamates and organophosphates were determined according to the protocols of Martinez et al. [17] and Weill et al. [18], respectively. The allelic frequency of these two mutations was evaluated at each site to analyse correlations with phenotypic resistance.

### Enzymatic tests on microplates

Approximately 30–50 *An. gambiae* (F1) females from each site, aged 2–5 days and not previously used for any insecticide test, were used for biochemical analyses. Before these analyses, these mosquito specimens were stored at −80 °C in dry microcentrifuge tubes. Biochemical enzyme assays [19] were carried out to compare the level of activity of mixed oxidases (MFO), non-specific esterases (α and β-esterases) and glutathione S-transferases (GST) in Parakou, Kandi and Malanville mosquito populations with that of *An. gambiae *sensu stricto (*s.s*.) Kisumu, a susceptible laboratory strain. Since enzymes degrade rapidly at room temperature, mosquitoes were ground on ice in 200 µl of distilled water and the extract was centrifuged at 12,000 rpm for 2 min. For GST, 10 µl of mosquito grindings in two replicates were put into each Nunc plate well to which 200 µl of a solution of reduced glutathione and 1-chloro-2,4-dinitrobenzene (CDNB)) was added. Concerning MFOs, after putting 20 µl of crushed material in two replicates in each well, 80 µl of 0.0625 M potassium phosphate buffer (KHPO4) pH = 7.2 and 200 µl of 0.25 M tetramethyl benzidine (TMBZ) solution pH 5.0 and, 25 µl of a 3% hydrogen peroxide solution were added in each well. For the non-specific esterases, 90 µL of two replicates of shredded material were added to each plate well, 90 µL of 1% triton phosphate buffer (PBS) pH 6.5, 100 µL of a solution composed of 0.3 M alpha-Naphthyl acetate (or beta-Naphthyl acetate) and Triton PBS pH 6.5, water and Fast Garnett Salt solution. Readings for each enzyme activity were taken as an endpoint at 340 nm, 630 nm and 550 nm for GST, MFO and non-specific esterases, respectively.

### Data analysis

The resistance status of malaria vectors was determined as follows:

- ≥ 98% mortality 24 h after insecticide exposure, the *An. gambiae* population is susceptible;
- Between 90 and 97% mortality 24 h after insecticide exposure, the *An. gambiae* population is suspected of being resistant (requires confirmation);
- < 90% mortality 24 h after insecticide exposure, the *An. gambiae* populations is resistant.

No mortality was recorded in controls. Therefore, Abbott’s formula was not necessary to correct the mortality rates.

The resistance status of the tested mosquito populations was determined according to WHO criteria [10]. The mortality rates of *An. gambiae* populations from the three different sites were compared using a stratified 2 × 3 contingency table and Pearson’s χ^2^-test in the statistical software, R 2.15. The strata in the 2 × 3 contingency table included the insecticide and the dosage. The allelic frequencies of the *kdr* L1014F and *ace-1* G119S genes were analysed to assess their variability across mosquito populations. Using SPSS® (SPSS Inc. Released 2009. PASW Statistics for Windows, Version 18.0. Chicago: SPSS Inc.), a comparative measure of mean enzyme activities between the study sites was performed to assess the variation in enzyme activity of mosquito populations in each locality using one-way analysis of variance (ANOVA). Tukey’s test was used to compare the means. Independent-samples t-test was performed to compare enzyme activity between field and laboratory (Kisumu) susceptible mosquitoes.

## Results

### Susceptibility testing of collected *Anopheles gambiae* to insecticides

A total of 2,501 female mosquitoes were exposed to insecticide-impregnated papers, of which 860 were from Malanville, 847 from Kandi, and 794 from Parakou (Tables 1, 2, 3). No mortality was recorded in the controls. Therefore, Abbott's formula was not used to correct the mortality rates.

**Table 1:** Mortality rate and resistance status of *Anopheles gambiae s.l.* from Malanville, Parakou and Kandi after exposure to papers impregnated with permethrin

District	Insecticide/Dose	Total tested	Mortality rate (%)	Nber dead/24 h	Nber alive	P-value	95% CI
Control	No	50	0	0	50		
Kandi	Per 1x	96	52^a^	50	46	˂0,0001	[41,6-62,4]
	Per 5x	100	92^b^	92	8		[84,8-96,5]
	Per 10X	99	99^c^	98	1		[94,5-100]
Malanville	Per 1x	93	16^a^	15	78	˂0,0001	[9,3-25,2]
	Per 5x	96	91^b^	87	9		[82,9-95,6]
	Per 10X	95	98^b^	93	2		[92,6-99,7]
Parakou	Per 1x	84	48^a^	40	44	˂0,0001	[36,6-58,8]
	Per 5x	87	84^b^	73	14		[74,5-90,9]
	Per 10X	84	99^c^	83	1		[93,5-100]

Mortality rate comparison by location which have the same letter do not differ significantly for the same dose in different populations at the 0.05 level using z-test; ^†^based on χ^2^-test

**Table 2:** Mortality rate and resistance status of *Anopheles gambiae s.l.* from Malanville, Parakou and Kandi after exposure to papers impregnated with deltamethrin

District	Insecticide/Dose	Total tested	Mortality rate (%)	Nber dead/24 h	Nber alive	P-value	95% CI
Control	No	50	0	0	50		
Kandi	Delta 1x	89	16^a^	14	75	˂0,0001	[8,9-25]
	Delta 5x	88	58^b^	51	37		[47-68,4]
	Delta 10x	96	100^c^	96	0		[96,2-100]
Malanville	Delta 1x	91	14^a^	13	78	˂0,0001	[7,8-23,2]
	Delta 5x	91	86^b^	78	13		[76,8-92,2]
	Delta 10x	93	96^c^	89	4		[89,4-98,8]
Parakou	Delta 1x	90	40^a^	36	54	˂0,0001	[29,8-50,9]
	Delta 5x	82	49^a^	40	42		[37,6-60,1]
	Delta 10x	96	73^b^	70	26		[62,9-81,5]

Mortality rate comparison by location which have the same letter do not differ significantly for the same dose in different populations at the 0.05 level using z-test; ^†^based on χ^2^-test

**Table 3:** Mortality rate and resistance status of *Anopheles gambiae s.l.* from Malanville, Parakou and Kandi after exposure to papers impregnated with bendiocarb

District	Insecticide/Dose	Total tested	Mortality rate (%)	Nber dead/24 h	Nber alive	P-value	95% CI
Control	No	50	0	0	50		
Kandi	Bendio 1x	87	94	82	5	0,4334	[87,1-98,1]
	Bendio 5x	95	96	91	4		[89,6-98,8]
	Bendio 10x	97	98	95	2		[92,7-99,7]
Malanville	Bendio 1x	98	97	95	3	0,1574	[91,3-99,4]
	Bendio 5x	102	99	101	1		[94,7-100]
	Bendio 10x	101	100	101	0		–
Parakou	Bendio 1x	87	98	85	2	0,742	[91,9-100]
	Bendio 5x	91	99	90	1		[94–100]
	Bendio 10x	93	99	92	1		[94,2-100]

Mortality rate comparison by location which have the same letter do not differ significantly for the same dose in different populations at the 0.05 level using z-test; ^†^based on χ^2^-test

**Fig. 2:**
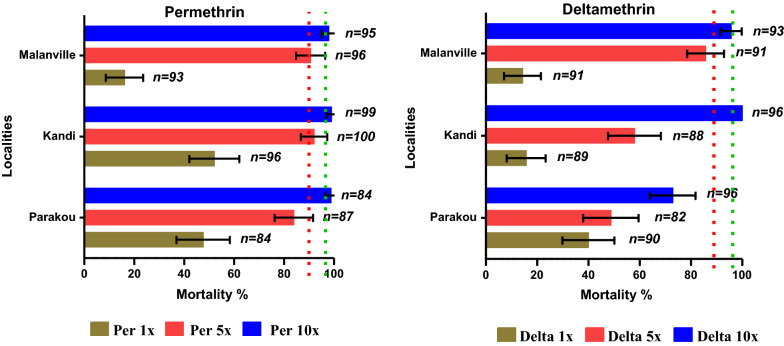
Mortality rate after exposure of *Anopheles gambiae* collected in Parakou, Kandi and Malanville to multiple doses of permethrin and deltamethrin diagnostic concentration, using WHO tubes bioassay

Pyrethroid resistance was recorded in all three communes with mortality rates below 60% (Table 1, Fig. 2). Of 91 mosquitoes in the Malanville population exposed to the diagnostic dose of deltamethrin (1x), only 13 were dead (14%) (Table 2, Fig. 2). When Malanville mosquitoes were exposed to higher 5 × and 10 × doses of deltamethrin, mortality rates increased from 14% (for the 1 × dose) to, respectively, 86 and 96% (Table 2, Fig. 2). Despite increasing the deltamethrin concentration by five times the diagnostic concentration (5x), only 86% of the tested mosquitoes were killed and more than 14% continued to fly 24 h after observation. Even with a 10 × concentration, not all mosquitoes were killed with deltamethrin (Table 2, Fig. 2). With permethrin 1x (diagnostic dose), only 16% were dead 24 h after the exposure time. With higher doses, 5 × and 10x, permethrin mortality rates increased from 16 to 91 and 98%, respectively, for mosquitoes in Malanville (Table 1, Fig. 2). The trends recorded in Malanville for these pyrethroids (deltamethrin and permethrin) (Tables 1 and 2) are the same in Kandi and Parakou also showing an increase in mortality rates of *An. gambiae* exposed to multiple diagnostic doses, but without killing all mosquitoes.

**Fig. 3:**
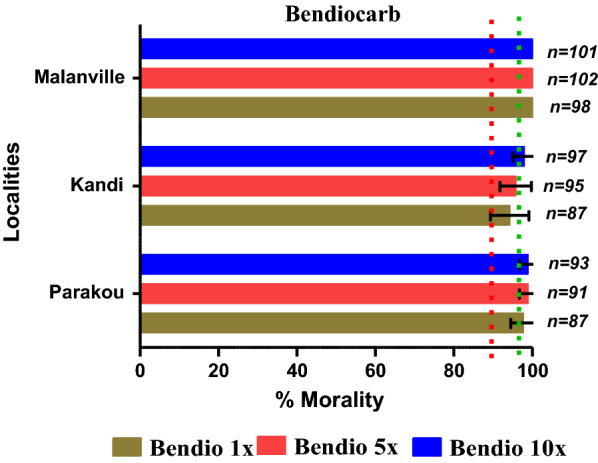
Mortality rate after exposure of *Anopheles gambiae* collected in Parakou, Kandi and Malanville to multiple doses of bendiocarb diagnostic concentration, using WHO tubes

For bendiocarb, suspected resistance was recorded in the communes of Malanville (97%) and Kandi (94%) while susceptibility was observed in Parakou (98%). When mosquitoes in Malanville were exposed to 5 × and 10 × higher doses of bendiocarb, mortality rates increased to 99 and 100%, respectively (Table 3, Fig. 3), reflecting the sensitivity of the Malanville populations to these doses. Similar results were recorded with mosquitoes from Parakou with a mortality rate of 99% for bendiocarb 5 × and 10x. However, with *An. gambiae* populations in Kandi, even a tenfold higher concentration did not kill all mosquitoes with a mortality rate of 98%.

There were significant mortality differences in Malanville, Kandi and Parakou at each individual insecticide-concentration combination of 1 × , 5 × , and 10 × deltamethrin, and 1 × permethrin (p < 0.001).

### Identification of *Anopheles gambiae* complex species and molecular characterization of *kdr* L1014F and *ace-1* G119S resistance alleles

Species PCR analysis detected the presence of three species of *An. gambiae* in Parakou including: *An. gambiae* s.s. (94%), *Anopheles coluzzii* (3%) and *Anopheles arabiensis* (3%). In Kandi, two species of this complex, namely *An. gambiae s.s.* (90%) and *An. arabiensis* (10%), were detected. (Tables 4 and 5).

**Table 4:** The frequency of the *kdr* resistant allele (L1014F) observed in the different *Anopheles gambiae* complex species

Localities	Species	Number	Genotypes			f (1014F)	χ^2^-value	df	p-value^*^
		Tested	*1014F*	*1014L*	*1014L*				
			*1014F*	*1014F*	*1014L*				
Kandi	*An. gambiae* s.s	90	58	25	7	0.78	0.0032	1	0.954
	*An. arabiensis*	10	6	3	1	0.75			
Parakou	*An. gambiae* s.s	94	64	23	7	0.80	0.720	2	0.697
	*An. coluzzii*	3	2	0	1	0.67			
	*An. arabiensis*	3	2	1	0	0.83			
Malanville	*An. coluzzii*	100	37	44	19	0.59	–	–	–

^*^p-value: based on χ^2^-square test comparing frequencies between the two species by locality; f = frequency

**Table 5:** The frequency of the *ace-1* resistant allele (G119S) observed in different *Anopheles gambiae* complex species

Localities	Species	Number	Genotypes			f (119S)	χ^2^-value	df	p-value^*^
		Tested	*119S*	*119G*	*119G*				
			*119S*	*119S*	*119G*				
Kandi	*An. gambiae* s.s	90	0	3	87	0.017	0.028	1	0.86
	*An. arabiensis*	10	0	1	9	0.050			
Parakou	*An. gambiae* s.s	94	0	5	89	0.027	0.327	2	0.84
	*An. coluzzii*	3	0	0	3	0.000			
	*An. arabiensis*	3	0	0	3	0.000			
Malanville	*An. coluzzii*	100	0	6	94	0.030	–	–	–

^*^p-value: based on the χ2-square test comparing frequencies between the two species by locality; f = frequency

The *kdr L1014F* mutation allelic frequencies ranged from 0.59 in Malanvile for *An. coluzzii* to 0.83 in Parakou for *An. arabiensis* (Table 4). For the *ace-1 G119S* mutation, the allelic frequencies were low ranging from 0.02 in Parakou to 0.05 in Kandi and Malanville (Table 5).

### Enzymatic tests on microplates

**Fig. 4:**
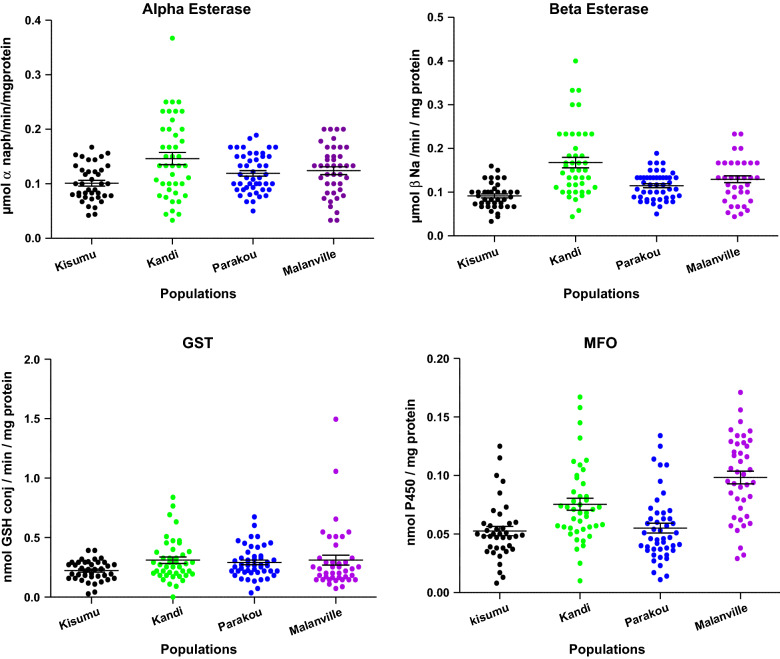
Mean and standard error of enzyme overexpression [α- and β- esterases, mixed-function oxidases (MFO) and glutathione-S-transferase (GST)] in *Anopheles gambi*ae *s.l.* in Kandi, Parakou and Malanville compared with *Anopheles gambiae s.s* Kisumu (laboratory reference strain) characterized by spectrophotometry

Enzyme activity in *An. gambiae* in the three communes was higher compared to the reference Kisumu strain (Fig. 4, Table 6). The activity of non-specific esterases (α and β esterase) was significantly higher in Kandi populations compared to the Kisumu strain (p < 0.05) (Fig. 4, Table 6). In Malanville and Kandi, significantly higher MFO levels were recorded in *An. gambiae* compared to the Kisumu strain (p < 0.05) (Fig. 4). In contrast to Kandi, no significant activity of MFO was recorded in Parakou. The activity of glutathione-S-transferases (GST) is very close to those of Kisumu in the three communes.

**Table 6:** Comparison and statistical analysis of the mean and standard error of the enzymatic activity of the mixed-function oxidases, glutathione-S-transferases, and esterases in *Anopheles gambiae* populations

Mosquito Population	MFO (Absorbance 630 nm)	GST (CDNA-ȵmol/min/mg protein)	α-Esterase (α-nmol/min/mg protein)	β-Esterase (β-nmol/min/mg protein)
Reference (Kisumu)	0.052 ± 0.004	0.222 ± 0.013	0.101 ± 0.005	0.091 ± 0.004
Kandi	0.075 ± 0.005^a,d^	0.310 ± 0.027^a^	0.146 ± 0.011^a,d^	0.167 ± 0.012^a^
Parakou	0.055 ± 0.004^b^	0.291 ± 0.019^a^	0.119 ± 0.005 ^b^	0.114 ± 0.004^a^
Malanville	0.098 ± 0.005^c,d^	0.310 ± 0.041^a,d^	0.124 ± 0.007 ^b^	0.129 ± 0.007^a^
One Way ANOVA	F = 20.16; df = 4; 166; p < 0.0001	F = 2.18; df = 4; 166; p = 0.009	F = 5.87; df = 4; 166; p = 0.0008	F = 16.52; df = 4; 166; p = 0.0001

*MFO* mixed function oxidases, *GST* glutathione-S-transferase;

Mean followed by a different letter were significantly different, P < 0.05, Tukey’s test

^d^Significant increase in mean differences compared to the laboratory reference strain, p < 0.05 using t-test

## Discussion

This study provides information about the intensity of insecticide resistance to deltamethrin, permethrin and bendiocarb in three communes of Benin. This study contributes to the knowledge base of insecticide resistance in Benin.

All three populations of *An. gambiae* showed high levels of resistance to the diagnostic dose of permethrin and deltamethrin, while suspected resistance was observed with bendiocarb in two study sites out of three. The pyrethroid resistance observed in the three communes confirms previous studies in Benin [3, 7, 20–22].

The lowest level of mortality to the diagnostic dose of permethrin was noted in Malanville (16%). Increasing the permethrin dose (5 × and 10 ×) increased the mortality rate, but not all mosquitoes exposed to permethrin-impregnated papers died within 24 h. The same was observed in Kandi and Parakou when *An. gambiae* populations were exposed to higher concentrations of permethrin (5 × and 10 × diagnostic dose). For deltamethrin, there were very low mortality rates in Kandi (16%) and Malanville (14%) compared to Parakou (40%). The use of 5 × and 10 × the diagnostic dose of deltamethrin showed strong resistance in Parakou with 49% mortality for the 5 × dose and 73% for the 10 × dose. Based on WHO guidelines [10], these results suggest that resistance levels in the three sites in northern Benin ranged from moderate to high for both deltamethrin and permethrin.

The *kdr* 1014F allele was found at very high frequencies in all tested *An. gambiae* populations, suggesting near fixation in the mosquito population. This spread of the *kdr* 1014F gene in malaria vector populations in Benin could compromise the effectiveness of the vector control tools that are currently in use [23, 24].

Biochemical data revealed over-production of MFOs in *An. gambiae* populations in Kandi and Malanville localities, which was similar to previous studies conducted in these areas [4, 5]. This finding is significant since oxidases are involved in the detoxification of pyrethroids in insects [25, 26].

Likely, the low mortality rates recorded with the diagnostic doses of pyrethroids in the three communes may be due to the very high insecticide selection pressure exerted on mosquitoes in these localities, characterized by massive cotton production and high use of chemicals to control pests. Additionally, ITNs and locally acquired mosquito avoidance pesticides (i.e., sprays and coils) may also contribute to the selection pressure. Resistance due to cotton cultivation in the north of the country has been reported by other authors [27, 28]. In Parakou, specifically, resistance could also be explained by the usage of insecticide products in market gardening. In addition to agricultural products, resistance may be further compounded by insecticidal pressures resulting from the massive use of LLINs [29, 30]. High selection pressure from LLINs and pervasive use of insecticides in agriculture has been implicated in the high oxidase levels in *An. gambiae s.s.* populations [29, 31]. Thus, the simultaneous presence of the *kdr* 1014F gene and elevated expression of oxidases may explain the high pyrethroid resistance intensities of malaria vectors observed in the two localities.

Given the status of pyrethroid resistance in these three areas, new generation LLINs treated with a pyrethroid and piperonyl butoxide (PBO) should be considered for distribution. Also, the use of nets impregnated with a pyrethroid combined with PBO may improve the effectiveness of LLINs in areas with high levels of MFOs [32, 33]. However, the potential limitation of this study was that synergistic bioassays with PBO were not conducted. It should be noted that the high GST activity found in wild populations of *An. gambiae* in Kandi may play a minor role in pyrethroid resistance due to detoxification of pyrethroid-induced lipid peroxidation products [34]. Suspected resistance to bendiocarb was also noted. This was previously observed in several localities in Benin [3, 7, 15]. However, the existence of mosquitoes surviving after exposure to doses five times higher than the diagnostic dose in Kandi may indicate that moderate resistance intensity to bendiocarb is developing in northern Benin. At the time of this study, bendiocarb was being considered for IRS in Benin. However, bendiocarb was not used for IRS in Benin in 2017, and the organophosphate pirimiphos-methyl was used instead.

Generally, *ace-1R* resistance alleles and esterases are associated with resistance to carbamates and organophosphates. In this study, *ace-1R* resistance allele frequencies in the three localities were 2.0 to 2.5% depending on the locality. Previous studies reported *ace-1R* resistance allele frequency of 0 to 1% [4, 15, 20, 35]. These results could indicate that the frequency of the ace*-1R* mutation is on the rise in Benin. However, the small sample size and the different locations used in this study from previous reports prevent confirming this increase in *ace-1R* with certainty. Recent results obtained by Aïkpon et al*.* in Atacora [3], Gnanguenon et al*.* in Kandi [15], and Salako et al*.* in Alibori and Donga [7] have also shown 1 to 6% in *ace-1R* allele frequencies in different areas of northern Benin. Moreover, the high activity of non-specific esterases noted in Kandi shows that bendiocarb resistance may not be linked to the presence of the *ace-1R* mutation only. Esterases can confer resistance to organophosphates and carbamates [34]. This overproduction of esterases is thought to be due to insecticidal pressure exerted on mosquito larvae in cotton crops to control the pests [22, 27]. This evolution of bendiocarb resistance coupled with the spread of the *ace-1* mutation allele in these mosquito populations could have serious implications for future IRS success. The insecticide rotation conducted during IRS in 2017 could curb the selection pressure of carbamate and organophosphate resistance in these populations and improve the effectiveness of pyrimiphos-methyl IRS in the area. However, it would be necessary to monitor the susceptibility of Anopheles to pyrimiphos-methyl and to find alternative vector control methods to curb the spread of resistance genes. Rotation or combination of insecticides in IRS are strategies to be promoted for resistance management, which is becoming an issue of concern.

## Conclusion

This study provides information on the intensity of resistance of malaria vectors to insecticides used in LLINs and IRS in Benin. These findings not only confirm the results of past studies of widespread loss of susceptibility to permethrin and deltamethrin but also show that the intensity of resistance is moderate to high in *An. gambiae* in different parts of Benin. This high intensity of resistance is a potential threat to vector control efficacy. The mechanisms of resistance may include the presence of the *kdr* L1014F gene and the overproduction of oxidases for the pyrethroids and increased activity of esterases for resistance to bendiocarb. Intensity results with pyrethroids suggest that alternatives to standard LLINs should be considered for use in northern Benin. Resistance assays with the insecticides alpha-cypermethrin, chlorfenapyr, and the synergist PBO should be routinely conducted as the scale-up of the distribution of new-generation nets (i.e., PBO ITNs or G2 ITNs) increases across sub-Saharan Africa. The results from these assays would serve as a guide in the choice of control strategies implemented by national malaria control programmes.

## Data Availability

The data used and/or analysed in this study are available from the corresponding author on reasonable request.

## Abbreviations

IRS: Indoor residual spraying
PMI: US President's Malaria Initiative
USAID: US Agency for International Development
WHO: World Health Organization
LLIN: Long-lasting insecticidal nets
NMCP: National malaria control programme
EIR: Entomological inoculation rate
PCR: Polymerase chain reaction
CREC: Centre de Recherche Entomologique de Cotonou
RTI: Research triangle international
ITN: Insecticide-treated net
CDC: Centers for disease control and prevention
MFO: Mixed function oxidases
GST: Glutathione S-transferases
CDNB: 1-Chloro-2,4-dinitrobenzene
TMBZ: Tetramethyl benzidine
ANOVA: Analysis of variance
PBO: Piperonyl butoxide
